# Does Place Matter When Understanding Loneliness and Social Isolation?

**DOI:** 10.1093/geroni/igab046.2205

**Published:** 2021-12-17

**Authors:** Annette Burns, Christina Victor, Thomas Prohaska, Brian Lawlor, Gerry Leavey, Roger O'Sullivan

**Affiliations:** 1 Institute of Public Health in Ireland, Dublin, Dublin, Ireland; 2 Brunel University London, Uxbridge, England, United Kingdom; 3 RRF Foundation for Aging, Fairfax, Virginia, United States; 4 Trinity College Dublin, Dublin, Dublin, Ireland; 5 Ulster University, Belfast, Northern Ireland, United Kingdom; 6 Institute of Public Health in Ireland, Belfast/Dublin, Dublin, Ireland

## Abstract

Physical distancing and restriction of movements as measures to prevent the spread of Covid-19 required people to change their work, home and social lives. Loneliness and social isolation have emerged as key public health issues during the pandemic. Traditionally when considering loneliness the focus is often on individual factors rather than within the context of structural and environmental dimensions. This paper will utilise data from the Coping with Loneliness, Isolation and Covid-19 global online survey which had over 20, 000 global responses from people aged 18+ in 2020. Analysis will use the lens of ‘place’ and the 5-item UCLA scale and 6-item Lubben social network scale to understand the social and demographic characteristics and structural and environmental factors associated with those experiencing loneliness and/or social isolation in rural and urban areas both before and during the pandemic. The paper will conclude with key messages from a public health perspective.

